# Influence of onion skin powder on nutritional and quality attributes of wheat pasta

**DOI:** 10.1371/journal.pone.0227942

**Published:** 2020-01-27

**Authors:** Monika Michalak-Majewska, Dorota Teterycz, Siemowit Muszyński, Wojciech Radzki, Emilia Sykut-Domańska

**Affiliations:** 1 Department of Plant Food Technology and Gastronomy, Department of Fruits, Vegetables and Mushrooms Technology, University of Life Sciences in Lublin, Lublin, Poland; 2 Division of Engineering and Cereals Technology, Department of Plant Food Technology and Gastronomy, University of Life Sciences in Lublin, Lublin, Poland; 3 Department of Biophysics, University of Life Sciences in Lublin, Lublin, Poland; University of Agriculture in Krakow, POLAND

## Abstract

Onion skin is a waste produced during onion bulb processing. Recent studies have reported that it contains large amounts of bioaccessible and bioavailable compounds thus it can be used to design of novel food products. The objective of the study was an attempt to substitute semolina with onion skin (OS) powder in pasta at 2.5, 5 and 7.5 g/100 g levels. The effects on the chemical composition, antioxidant potential, technological and sensory properties of the fortified pasta samples were evaluated compared with a control sample. Fortification with OS resulted in a significant (P < 0.05) improvement in nutritional properties, which was demonstrated by an increase in the content of dietary fibre, ash, total phenolic compounds, flavonoids content and antioxidant activity (FRAP and DPPH). Cooking loss increased with increasing levels of OS, however, all pasta samples were in the acceptable range (8 g/100 g). Onion skin incorporation decreased optimal cooking time, water solubility index and increase redness (a*), compared to the control sample. Results of sensory evaluation suggest that pasta, in which 2.5% of the flour was replaced by this plant component, showed the highest value of the “overall quality”. Our study indicates that onion skin powder can be a potential alternative for the food industry to provide nutritional enriched pasta.

## Introduction

In many countries, pasta is the second most consumed cereal product, just after bread. World pasta consumption is the largest in Italy (25.3 kg per capita), because pasta is the pillar of the Mediterranean diet [1]. Poland with an average annual consumption of about 5 kg per person, is nearly 20th in this ranking. The unquestionable advantages of pasta, such as ease of preparation, long shelf life and relatively low price, make its consumption gradually increased. There is also a growing assortment of pasta products, differing in shape and raw material composition [2]. The volume of pasta consumption makes it suitable for the use as a carrier of bioactive substances. Currently, many studies focused on the incorporation of various ingredients into pasta, including: chia flour [3], onion [4], moringa leaves [5], legume flours [6], and mushrooms [7], or fish [8, 9]. Similarly attempts have been taken to prepare nutrient-rich pastas fortified with different kinds of vegetable by-products: root vegetable pomace [10], outer bracts, leaves and stems of artichoke [11], and mango peel [5]. This kind of fortification affects pasta quality, in terms of texture, color, technological quality and sensory properties.

In recent years, demand for processed onions has increased, which has led to an increase in the amount of waste production (more than 500 000 tons each year). The main onion by-products are: dry skins, two outer fleshy scales and roots (generated during industrial peeling), and undersized, malformed, diseased or damaged bulbs. These wastes represent an environmental problem, due to the fact that are prone to microbial spoilage, which limits its further exploitation. Onion wastes, e.g. fleshy scales, are not suitable for fodder in high concentrations, due to the onions characteristic aroma. Also other waste are not useful for an organic fertilizer because of the rapid development of phytopathogenic agents [12]. It should also be noted that any kinds of plant by-products should be subject to effective quality control systems to excluding the presence of toxins such as solanine, patulin, ochratoxin, dioxins and polycyclic aromatic hydrocarbons. Afterwards such materials may be used for further processing [13].

In the literature there are a number of suggestions for the processing of onion by-products. Several studies considered the influence of pressure cooking, divalent cations and extrusion cooking on cell-wall polymers of onion waste [14, 15, 16]. Two outer fleshy leaves have been demonstrated as the most suitable sources to the recovery of fructan and fructooligosaccharides (FOS) [17]. The production of natural food colorants, alcohol, snacks and extrudates from onion pomace has also been reported [18, 19, 20, 21, 22]. A possible way of utilizing at least the onion skin may be to transform it into a biologically high-value functional ingredient. It seems appropriate, because this part of onion contains a number of bioavailable compounds with a documented high nutritional value. In particular: dietary fibre (DF), FOS, quercetin aglycones, minerals and small quantities of alk(en)yl cystein sulfoxides (ACSOs) [12, 23]. In rat model study, it was investigated that the onion by-products have multidirectional beneficial effects on the functioning of an organism [24].

Previous studies have shown that dry onion may be added to bakery products (rolls, breads), causing significant improvement of antioxidants abilities without the loss of its sensory properties [25, 26]. However, the physicochemical properties of pasta with partial replacement of semolina by onion skin powder have not yet been investigated. Thus, the aim of this study was to evaluation of substituting semolina flour with onion skin powder at various concentrations and study the changes in nutritional, technological and sensorial characteristics of the pasta.

## Materials and methods

### Materials

Material used in the study consisted of semolina durum (SE) (Jula Malom, Kunszállás, Hungary) and onion of Polanowska cultivar (Czesławice Experimental Farm, 51°18′23″N, 22°16′02″E, Poland). All chemicals reagents were of analytical grade, and were acquired from Sigma Aldrich (Switzerland).

### Onion skin (OS) powder preparation

Onion was manually peeled to obtain dry skin, that was washed twice with deionized water and dried in convection dryer (Zelmer, Poland) at 50 ^o^C for 12 h. Dried material was powdered using a laboratory mill (Proficook, Poland) and then sieved to pass through the appropriate 0.5 mm mesh screen.

### Pasta production

Pasta was prepared with semolina (SE), which was replaced with onion skin (OS) at 0%, 2.5%, 5%, 7.5% levels (CO, OS2.5, OS5, OS7.5, respectively). Pasta was produced in a semi-industrial scale line (MAC 30S, ItalPast, Fidenza, Italia, screw speed 60 rev/min, extrusion pressure 15 MPa). SE, with the addition of OS, was hydrated to reach 30% moisture, mixed in a vacuum mixer and extruded at low temperatures (die temperature 40°C). In the production of pasta, teflon (PTFE) dies were used. Then pasta was dried in static dryer (EAC 30-E, ItalPast, Fidenza, Italia) with 35–55°C and the humidity level of 85–55%. Dried pasta (100 g) was cooked in 1000 mL of boiling distilled water, each for the experimentally determined optimum time. After cooking, the pasta was cooled at room temperature.

### Chemical composition of pasta

The ash, moisture, total protein and total fat in uncooked and cooked pasta was determined according to AACC 08–01, 44-15A, 46–13, 58–19 [27]. To determine the dietary fibre (TDF) and its fractions (insoluble (IDF), and soluble (SDF) fibre), an enzymatic methods were used: AACC 32–05, 32–21 [27], AOAC 991.43, 985.29 [28]. The total carbohydrate content was estimated by subtracting the protein, fat, ash and moisture content from 100%. Available carbohydrates was calucalted by subtracting total dietary fibre from the total content of carbohydrates. The energy valve was calculated using the formula described by FAO, using the Atwater factors (single factor for each of the energy-yielding substrates). The energy values are 4.0 kcal/g for protein, 9.0 kcal/g for fat, 4.0 kcal/g for carbohydrates and 2.0 kcal/g for dietary fibre [29].

Energy value (kcal/100 g) = 4 x protein (g) + 9 x fat (g) + 4 x carbohydrate (g) + 2 x dietary fibre (g)

### Antioxidant properties of pasta and constituents

#### Total polyphenol content (TPC)

TPC was determined according to the method by Singleton and Rossi [30] with some changes [31]. The amount of TPC was expressed as gallic acid equivalent (GAE) in mg per g of dry matter (d.m.)

#### Total flavonoids content (TFC)

In previously prepared extracts, the total flavonoids content (TFC) was determined by the method of Zhishen et al. [32]. The result was expressed as mg of quercetin (QE) per 1 g of dry matter (d.m.).

#### Determination of antioxidant capacity

Antioxidant capacity was determined by scavenging ability of 1,1-diphenyl-2-picrylhydrazyl (DPPH) radicals according to the method of Brand-Williams et al. [33] with slight modifications [31]. The reducing power was also measured by the ferric reducing antioxidant potential (FRAP) assay [34]. To prepare the standard curve, Trolox was used and the results were expressed as mmol of Trolox equivalent (TE) per 1 g of dry matter (d.m.).

### Technological quality of pasta

#### Optimal cooking time (OCT) and cooking losses

Optimal cooking time (OCT) and cooking losses were determined according to the AACC 66–50 [27].

#### Weight increase index and volume increase index

Weight increase index and volume increase index of the pasta were determined according to procedure described by Sobota et al. [35].

#### Water solubility index (WSI) and water absorption index (WAI)

Water solubility index (WSI) and water absorption index (WAI) were determined using centrifuge method according to the AACC 56–20 [27].

### Color measurements

The color of raw materials and pasta products (uncooked and cooked) was determined using a 8200 Series reflection spectrocolorimeter (X-Rite) with a D65 illuminant according to the method described by Hunt [36] and Hunter & Harold [37]. Color readings were taken from five separate points on the samples. Results were expressed as L* (lightness), a* (redness) and b* (yellowness). The change in color due to onion skin powder fortification was determined by calculating the color differential index (ΔE) applying equation as previously [9].

### Sensory analysis

Sensory evaluation of uncooked and cooked pasta samples was carried out by a group of panelists consisted of 19 trained people (aged between 24 and 39 years old), who have experience in the area of estimation and definition of pasta parameters. The panelists participated in a series of screening tests to determine their level of skill, as recommended by ISO [38]. Analyses were carried out under conditions and according to the rules described in the study by Stone & Sidel [39]. The panelists were asked to indicate: color, odor and overall quality of the uncooked pasta, and in addition taste, hardness, adhesiveness and springiness of cooked samples. Hardness was the resistance of cooked pasta to compression by the teeth. Adhesiveness was evaluated by placing the pasta in the mouth, pressing it against the palate and determining the force required to remove it with the tongue. Springness was measured as the degree to which the product returns to its original shape after partial compression between the tongue and palate [35]. To quantify each parameters, a 9-point scale, from extremely unpleasant (1), through acceptable (5), to extremely pleasant (9), was used.

### Ethics

Only adults participated in the sensory study of uncooked and cooked pasta. The Local Bioethics Committee in Lublin, Poland, concluded that the above study did not require the consent of the Commission. These studies did not have a predictable risk or discomfort, nor did they expose participants to pain and no personal or identifying information was collected and all data were analysed anonymously. As such, while written participant consent was not collected for this study, all participants gave verbal informed consent to participate. They were informed about the nature of study, its objectives as well as participants’ confidentiality and anonymity. To make participants feel comfortable, they were allowed to withdraw from the study at any time and for any reason. All participant evaluated the tested products objectively and agreed to publish their evaluation results anonymously.

### Statistical analysis

All chemical and technological analyses were performed in triplicate. Data obtained during the study were subjected to one-way analysis of variance (ANOVA) and significance difference in the response and sample were evaluated by Tukey's comparison test (P < 0.05). Between selected parameters Pearson's correlation coefficients were also calculated. Statistical analyses were performed using Statistica 10 software (Stat-Soft, Poland).

## Results and discussion

### Proximate chemical composition

Raw material (SE and OS) composition used for the preparation of pasta product affects the physical, chemical and textural properties of a final product [40]. Characteristics of raw materials for the production of various pasta types is presented in the following table (Table 1).

**Table 1:** Characteristics of raw materials for the production of fortified pastas.

Parameters	SE	OS
**Moisture [% d.m.]**	9.49 ± 0.04	9.89 ± 0.10
**Protein [% d.m.]**	12.96 ± 0.84	2.58 ± 0.10
**Fat [% d.m.]**	1.47 ± 0.05	0.77 ± 0.03
**Ash [% d.m.]**	0.89 ± 0.02	5.50 ± 0.18
Carbohydrate *[% d.m.]	70.59 ± 0.92	19.17 ± 0.99
**TDF [% d.m.]**	4.59 ± 0.19	62.09 ± 0.77
**IDF [% d.m.]**	2.13 ± 0.09	54.71 ± 0.62
**SDF [% d.m.]**	2.46 ± 0.10	7.38 ± 0.59
**Energy [kcal/100 g]**	356.61± 0.51	218.10 ± 2.22
**Total polyphenol [mg GAE/g d.m.]**	0.45 ± 0.02	34.73 ± 0.60
**Total flavonoids [mg QE/g d.m.]**	0.15 ± 0.02	41.81 ± 4.83
**DPPH [mm TE/g d.m.]**	1.86 ± 0.18	131.53 ± 2.09
**FRAP [mm TE/g d.m.]**	1.36 ± 0.07	274.74 ± 2.20
L*	91.04 ± 0.49	63.93 ± 3.34
a*	1.43 ±0.10	16.41 ±1.36
b*	21.05 ± 0.42	31.95 ± 3.38

SE–semolina durum; OS–onion skin powder; other abbreviations are explained in the methodology

* available carbohydrates, calucalted by substracting total dietary fibre from the total content of carbohydrates

The onion skin (OS) powder incorporation decreased (P < 0.05) the protein, fat, carbohydrate and moisture content (the latter value only in cooked pasta), whereas increased (P < 0.05) the ash and dietary fibre content (Table 2), potentially due to the OS composition (Table 1). Statistically significant decrease in the moisture content of cooked pasta with increase in OS can be attributed to a greater protein-polysaccharides interaction when compared to the control [9]. Previous research has also shown a decrease in protein and carbohydrate contents when onion skin powder was added to noodles [41]. In other studies, in which pasta was enriched with carrot powder and tomato peel flour, protein concentrate was lower than in control sample, as well [5]. This is understandable, because these ingredients are not high-protein sources. The absence of gluten proteins in that materials makes it difficult to form a sufficiently strong protein matrix that binds and closes the starch granules. This results in a lower culinary quality of that pastas, which is manifested by certain losses of dry matter during cooking, susceptibility to overcooking, lower firmness and elasticity of these products after cooking [40].

**Table 2:** Chemical composition and energy value of pasta fortified onion skin powder.

Sample	Parameters								
	Moisture	Protein	Fat	Ash	Carbohydrate *	TDF	IDF	SDF	Energy
	% d.m.								kcal/100 g
**Uncooked pasta**									
**CO**	8.29 ± 0.04 ^a^	12.56 ± 0,86 ^b^	1.42 ± 0.05 ^e^	0.89 ± 0.02 ^c^	72.12 ± 0.76 ^e^	4.73 ± 0.16 ^a^	2.20 ± 0.06 ^a^	2.53 ± 0.10 ^ab^	360.95 ± 0.29 ^f^
**OS2.5**	9.52 ± 0.53 ^b^	12.48 ± 0.87 ^b^	1.35 ± 0.03 ^de^	0.90 ± 0.01 ^c^	69.20 ± 0.91 ^d^	6.54 ± 0.18 ^b^	3.36 ± 0.11 ^b^	3.18 ± 0.07 ^e^	352.00 ± 2.06 ^e^
**OS5**	9.35 ± 0.39 ^b^	12.12 ± 0.85 ^b^	1.30 ± 0.05 ^cd^	1.12 ± 0.01 ^e^	67.18 ± 1.25 ^cd^	8.94 ± 0.07 ^c^	5.55 ± 0.13 ^d^	3.38 ± 0.06 ^d^	346.75 ± 1.27 ^d^
**OS7.5**	9.33 ± 0.03 ^b^	11.81 ± 0.78 ^b^	1.23 ± 0.05 ^c^	1.33 ± 0.01 ^f^	65.88 ± 0.77 ^c^	10.42 ± 0.13 ^d^	6.25 ± 0.14 ^e^	4.17 ± 0.01 ^f^	342.73 ± 0.09 ^c^
**Cooked pasta**									
**CO**	62.54 ± 0.21 ^e^	5.49 ± 0.36 ^a^	0.61 ± 0.02 ^b^	0.73 ± 0.02 ^a^	25.82 ± 0.49 ^b^	4.92 ± 0.06 ^a^	2.49 ± 0.11 ^a^	2.43 ± 0.04 ^a^	141.54 ± 0.61 ^b^
**OS2.5**	62.02 ± 0.10 ^de^	5.11 ± 0.24 ^a^	0.59 ± 0.02 ^ab^	0.87 ± 0.05 ^b^	24.63 ± 0.40 ^b^	6.83 ± 0.17 ^b^	4.22 ± 0.11 ^c^	2.61 ± 0.07 ^bc^	137.94 ± 0.54 ^b^
**OS5**	61.63 ± 0.14 ^cd^	5.00 ± 0.31 ^a^	0.52 ± 0.02 ^a^	1.03 ± 0.01 ^d^	21.64 ± 0.69 ^a^	10.20 ± 0.23 ^d^	7.45 ± 0.18 ^f^	2.75 ± 0.05 ^c^	131.59 ± 0.99 ^a^
**OS7.5**	61.10 ± 0.08 ^c^	4.93 ± 0.20 ^a^	0.51 ± 0.01 ^a^	1.07 ± 0.04 ^d^	21.19 ± 0.31 ^a^	11.23 ± 0.12 ^e^	8.17 ± 0.14 ^g^	3.05 ± 0.02 ^d^	131.49 ± 0.57 ^a^

* available carbohydrates, calucalted by substracting total dietary fibre from the total content of carbohydrates

CO–control sample: pasta prepared with 100% of semolina flour; OS2.5, OS5 and OS7.5: pasta prepared with 2.5, 5, 7.5 g of onion skin powder/100 g of semolina flour.

Means with different letters in the same column are significantly different (P < 0.05), ± standard deviation for three replicate determinations.

With the addition of OS, the ash content of the pasta samples increase (Table 2), which was caused by high ash content of OS use in our study (5.5% d.m.) (Table 1). In studies of Sayed et al. [41] ash content in noodles also increased with the addition of onion skin powder, which contained similar amount of ash. It was found that brown skin of onion is the industrial waste with superior total ash, the content of which decreases from the outer to the inner part of the onion bulb (from 10.6 to 4.7% d.m., respectively) [12]. Like in Desai et al. [9] studies, the ash content of cooked pasta samples was lower than that of uncooked samples. The greatest decrease in the ash content was observed for sample with the highest content of onion skin (OS7.5), which may be caused by higher cooking losses.

**Fig 1:**
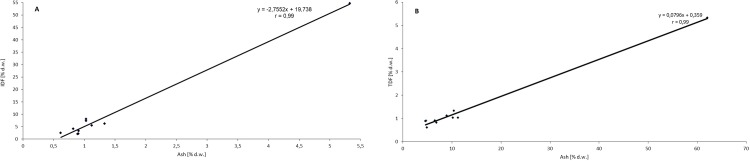
Relationship between the contents of ash–total dietary fibre (A) and ash—insoluble dietary fibre (B) in pasta fortified with onion skin powder.

Onion is an important source of total dietary fibre (TDF) showing a better soluble:insoluble dietary fibre ratio (SDF:IDF) than other vegetables, which is connected with different metabolic and physiological effects. In addition, onion skin contains the most dietary fibre of its all tissues, and it is dependent on the cultivar [12, 42]. OS used in the study contains 62.09% TDF in dry matter, including 54.71% IDF and 7.38% SDF (Table 2). These results are in agreement with other authors, who showed nearly the same SDF:IDF ratio (1:8). It is worth pointing out that in brown skin, SDF:IDF ratio is similar to that of cereal bran, whereas in inner scales, this ratio is near to that observed in some fruit by-products [43]. The IDF fraction consists mainly of cellulose and hemicelluloses, whilst the soluble fraction contains mainly pectins [34]. After addition of OS, content of TDF increases significantly in samples (P < 0.05), both in the uncooked and cooked pasta (Table 2). Furthermore, in the cooked samples, the content of TDF and IDF is higher, compared to the uncooked sample. Sobota et al. [35] states that the increase in the IDF content may be due to an increase in the content of resistant starch in pasta after cooking. Observed changes may also be caused by the fact that the individual ingredients of dry matter are washed out disproportionately during cooking. It is reasonable to assume that the components of IDF migrate less than the components of SDF into the water during cooking. The IDF content of dry matter of the cooked pasta is therefore increased. In the same way, the decrease in the SDF content of the cooked samples as compared to the uncooked samples of pasta, can be explained. Our studies indicated that the ash content in pastas fortified with onion skin is positively correlated with the total dietary fibre content (r = 0.99), including mainly the insoluble fraction of fibre (r = 0.99), Fig 1A and 1B.

Pasta incorporation with OS powder showed reduction of energy value of samples, with the increase in concentration of OS. However, no difference was observed between pairs of cooked pastas (CO—OS2.5 and OS5—OS7.5), about 139.5 and 131.5 kcal/100 g, respectively (Table 2). The reduction in energy value of pasta enriched with OS could be due to the increase in the content of ash and each fraction of fibre, and a decrease in the concentration of carbohydrates and fat, almost absent in the OS (Table 1).

### Total polyphenol content (TPC), total flavonoids content (TFC) and antioxidant properties of pasta (DPPH, FRAP)

**Fig 2:**
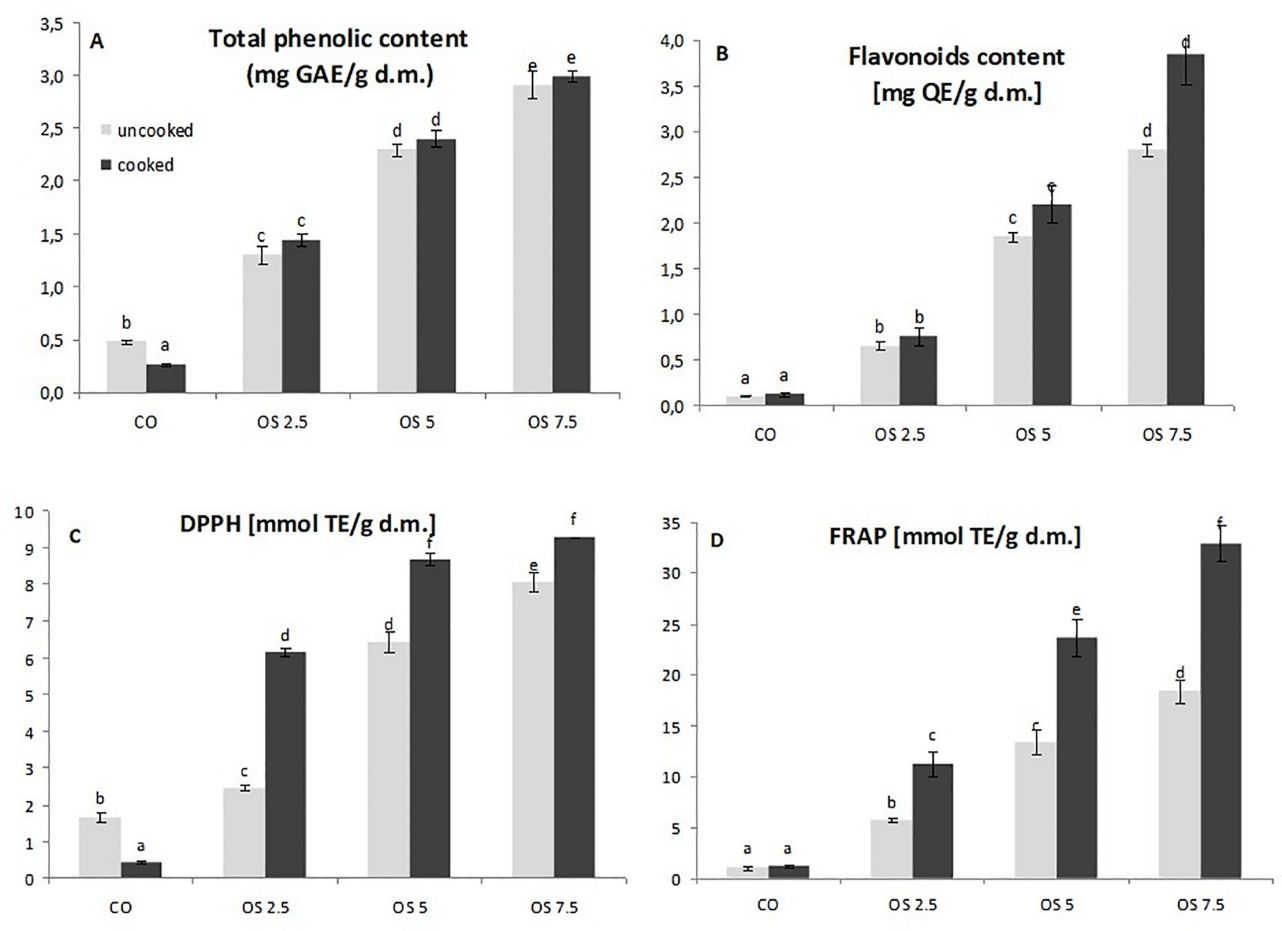
Total polyphenol content (A), total flavonoids content (B), antioxidant capacity by DPPH (C) and FRAP (D) of pasta fortified with OS. Bars are the mean ± SD of three values. Different letters indicate significant difference (P < 0.05).

The results of TPC and TFC analysis show that the addition of OS powder significantly increased the total phenolic and flavonoids content, for both uncooked and cooked pasta, reaching the highest value in cooked OS7.5 (Fig 2A and 2B). While the TPC is decreased in the cooked CO, TPC of all the pasta-OS after cooking has not decreased. This may suggest that antioxidant compounds have not been disturbed and their content in pasta shows an increase, although not statistically significant. Regarding the antioxidant capacity measured by DPPH and FRAP, the tendency is the same for uncooked and cooked pasta, showing an increase of activity directly correlated with the higher OS content (Fig 2C and 2D). Our results correspond to previous studies upon pasta fortified with phenolic-rich materials, such as chia flour [3], mushroom powder [7], or noodles enriched onion skin powder [41]. As well as baked rolls containing dried onions, in which positive relationships between TPC, antioxidant capacity and the proportion of added materials, have been observed [26].

The DPPH and FRAP studies also mean that there is a significant release of phenolic constituents when cooking pastas with OS. A similar trend was observed during cooking pasta with different levels of partially de-oiled chia flour [3]. These observations confirm a statement from other studies showing that the cooking process enhances the antioxidant properties of wheat pasta, which could be explained by the release of some phenolic acids from wheat caused by high temperatures [44].

It can be presumed, that phenolic compounds released during the boiling process are most probably components from OS, and not those provided by SE (Table 1). This is confirmed by higher antioxidant potential (DPPH, FRAP) as well as significantly higher content of flavonoids (TFC) in cooked pasta with OS (Fig 2B–2D). These results correspond to Sayed et al. [41] who found that the total phenolic, total flavonoids and DPPH of noodles with onion skin powder was significantly higher than in the control. The 6% of it addition caused improvement of total flavonoids to 2.37 mg/g. In other studies, the retention of quercetin (as a representative of onion flavonoids) was also evaluated. It is very important in terms of nutritional properties of pasta enriched with a component that is expected to improve the pro-health properties of the final product [4]. Currently, there are many *in vitro* and *in vivo* studies focused on onion, especially its skin, as a good source of food ingredients (including quercetin and its derivatives) that exhibit different mechanisms of action beneficial for health [23, 24].

### Effect of OS inclusion on technological quality of pasta

Cooking quality of pasta is the most important consumer attribute, including parameters such as cooking time, cooking loss, water solubility index and water absorption index [35, 40]. Values of these parameters in relation to our samples are presented in Table 3.

**Table 3:** Technological properties of pasta fortified onion skin powder.

Sample	Optimal cooking time (min)	Weight increase index	Volume increase index	Cooking loss (%)	WSI (%)	WAI (%)
**Uncooked pasta**						
**CO**	-	-	-	-	16.90 ± 0.04 ^h^	170.52 ± 1.86 ^b^
**OS2.5**	-	-	-	-	15.54 ± 0.04 ^g^	164.21 ± 3.17 ^b^
**OS5**	-	-	-	-	15.07 ± 0.08 ^f^	145.02 ± 2.82 ^a^
**OS7.5**	-	-	-	-	14.82 ± 0.07 ^e^	133.86 ± 2.84 ^a^
**Cooked pasta**						
**CO**	8.00 ± 0.50 ^b^	2.40 ± 0.02 ^b^	2.59 ± 0.02 ^b^	4.68 ± 0.11 ^a^	6.32 ± 0.10 ^d^	253.38 ± 2.82 ^d^
**OS2.5**	7.00 ± 0.26 ^a^	2.38 ± 0.06 ^ab^	2.68 ± 0.01 ^c^	5.47 ± 0.05 ^b^	5.57 ± 0.07 ^c^	247.15 ± 3.63 ^cd^
**OS5**	6.30 ± 0.26 ^a^	2.34 ± 0.06 ^ab^	2.66 ± 0.02 ^c^	5.91 ± 0.06 ^c^	5.10 ± 0.07 ^b^	250.86 ± 4.69 ^cd^
**OS7.5**	6.30 ± 0.40 ^a^	2.28 ± 0.02 ^a^	2.54 ± 0.02 ^a^	6.33 ± 0.06 ^d^	4.74 ± 0.04 ^a^	236.10 ± 1.18 ^cd^

CO–control sample: pasta prepared with 100% of semolina flour, OS2.5, OS5 and OS7.5: pasta prepared with 2.5, 5, 7.5 g of onion skin powder/100 g of semolina flour.

Means with different letters in the same column are significantly different (P < 0.05), ± standard deviation for three replicate determinations.

The optimum cooking time for the pasta decreased from 8.0 min (CO) to 7.0–6.5 min when the OS was added. A similar trend was observed in Sobota’s et al. [35] studies for pasta with wheat bran addition, Desai’s et al. [9] for pasta with fish powder, or Aranibar et al. [3] for pasta with chia flour. Observed changes may be caused by a disturbance of the gluten matrix caused by added fibre particles, which provided a path for water absorption into the semolina and also affected the shortening of cooking time. Shorter cooking time of pasta can also result in a lower increase in weight after cooking. This is because shorter cooking times reduce the water absorption of pasta.

Lower value of the weight increase index can also be caused by the increased TDF content of pasta with the addition of OS. Aravind et al. [45] state that food containing bran usually absorbs less water. Both bran and onion skin, consist mainly of cellulose and hemicelluloses. OS, like bran, can compete with starch for water during cooking [45], which results in lower weight increase index for pasta samples. According to Dick and Youngs [46], high quality pasta's weight increases threefold after cooking. The obtained pasta samples have the weight increase index ranging from 2.28 (OS7.5) to 2.40 (CO) (Table 3).

**Fig 3:**
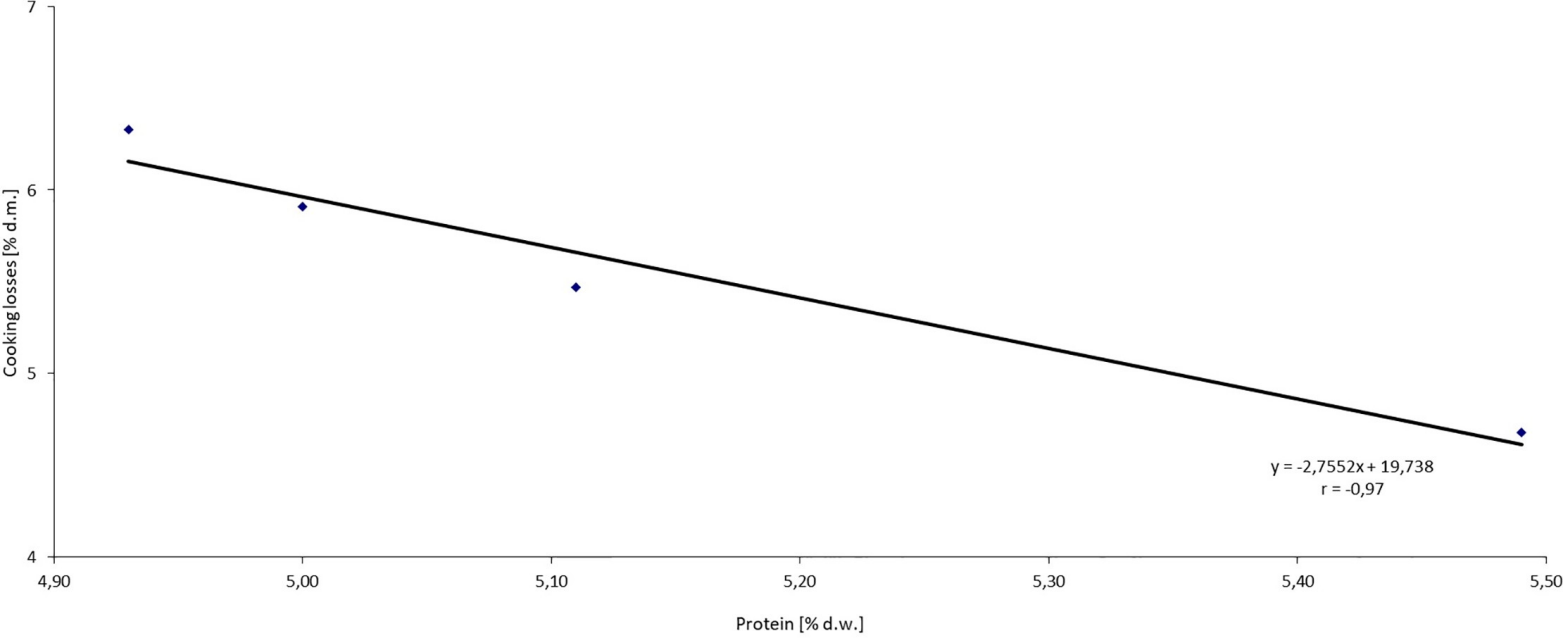
Relationship between the protein content and cooking losses of dry matter in pasta fortified with onion skin powder.

One of the most important parameters determining the quality of pasta is the cooking loss, which should not exceed 8% d.m. [46]. In the pasta fortified with OS, dry matter losses during cooking ranged from 4.68 to 6.33% [Table 3] and were negatively correlated with protein content in these products (r = -0.97), Fig 3. The highest cooking loss was recorded for sample OS7.5 (6.33%) and the lowest for CO (4.68%), which is an evidence of high culinary quality of pasta. Cooking losses increased with the addition of OS. A similar trend was observed by Sayed et al. [41] for noodles with onion skin powder addition. Manthey and Schorno [47] argue that the bran and bran-like particles, like OS, may inhibit the formation of gluten matrix during the pasta molding process. Water penetrates more easily the pasta structure during cooking, and starch granules are leached out more readily. Presence of large particles of bran-like ingredients can further disintegrate pasta dough by disturbing the even moistening of the material and inhibiting the gluten matrix formation [35].

According to Fuad and Prabhasankar [40], good-quality pasta should increase its volume three to four-fold after cooking. Tested samples had this index ranging from 2.54 (OS7.5) to 2.68 (OS2.5). After cooking, the volume of pasta for samples OS2.5 and OS5 increased in comparison with the control sample, but when this addition was exceeded, the index started to decrease. This may be due to the fact that pasta with OS swells and increases its volume by absorbing water, but when a certain amount of the OS in pasta is exceeded, it damages the structure of dough to such an extent that the pasta can no longer absorb large amounts of liquid. Sayed et al. [41] observed that onion skin powder addition increased linearly the volume of fried noodles. Large-volume food products are very important in a diet. Studies have shown that increasing the volume of food by increasing water addition, during cooking, reduces the energy density of the meal and subsequently, the energy consumption [48].

The addition of OS resulted in a significant decrease in the value of WSI in uncooked and cooked pasta (Table 3). Low value of WSI in pasta with OS may have been influenced by high content of insoluble fibre. The WSI index refers to the dry substance solubility in water. Products with low WSI are slowly digesting in the gastrointestinal tract. Low dry matter solubility may also indicate low glycemic index and low post-meal glycaemia [35]. WAI is a continuation of the WSI designation. The WAI value decreases significantly with the addition of OS for uncooked pasta from 171 (CO) to 134% (OS7.5). A decrease in WAI for cooked pasta was also observed, but it was not statistically significant (236–253%). Cooked pasta had higher WAI index than uncooked pasta. The WAI is closely linked to the degree of gelatinization of the starch. Gelatinized starch absorbs more water than native starch, which results in higher water absorption of cooked pasta. In nutrition, a high water absorption of food products is desirable because of reduction in the energy density of the meal. Products with high water absorption index swell in the stomach, which makes them more effective in satisfying hunger and giving the feeling of satiety [35].

### Effect of OS inclusion on color of pasta

Color is one of the most important quality properties for the acceptability of food due to its relation with product freshness and flavor expectations. Over the past few years, pasta is available in a wide range of colors, from traditional yellow shades to red, green and black. The color is the result of synthetic colorants or, increasingly often, the addition of extracts from plants (carrot, tomato, beet, spinach, herbs, microalgae), fungi (*Monascus purpureus*) or fish [2, 9].

Table 4 shows the L*, a* and b* values for all pasta samples before and after cooking. Raw and cooked samples enriched with OS powder (OS2.5, OS5, OS7.5) showed lower lightness (L*) value than control pasta (CO). After cooking, the coloring of the pasta became lighter. It was best observed in the sample with addition of 7.5 g OS/100 g semolina flour (P < 0.05). This trend for change is due to addition of OS powder, which is slightly brown in color. We also have reported similar observation in our study when onion powder was added to rolls [26]. In addition, Rajeswari et al. [4], who added different hydrocolloids to improve the quality characteristics of pasta with addition of 10% onion powder, reported that each of the obtained pasta products was characterized by decreased lightness (L*) value compared to the control (durum semolina alone). In other studies, where semolina was enriched with various additives (wheat bran, powder from fish, Spirulina), an increase in darkness (L*) in the color of pasta products was also observed as the increased concentration of substances added [9, 35].

**Table 4:** Color characteristics of raw material and pastas fortified onion skin powder.

Sample	Measurements			
	L^*^	a^*^	b^*^	*ΔE*
**Uncooked pasta**				
**CO**	74.90 ± 1.65 ^de^	3.34 ± 0.08 ^a^	37.72 ± 2.28 ^c^	
**OS2.5**	55.19 ± 4.95 ^b^	18.71 ± 1.54 ^d^	33.04 ± 3.99 ^bc^	25.63 ± 2.27 ^ab^
**OS5**	55.27 ± 1.21 ^b^	22.13 ± 0.05 ^e^	34.03 ± 0.94 ^c^	27.45 ± 0.76 ^b^
**OS7.5**	43.98 ± 1.60 ^a^	22.85 ± 2.24 ^e^	33.83 ± 3.09 ^c^	36.82 ± 1.17 ^c^
**Cooked pasta**				
**CO**	77.60 ± 2.04 ^e^	2.07 ± 0.34 ^a^	27.95 ± 0.67 ^b^	
**OS2.5**	70.68 ± 0.91 ^d^	6.29 ± 0.22 ^b^	15.24 ± 0.23 ^a^	18.26 ± 2.10 ^a^
**OS5**	63.89 ± 1.73 ^c^	12.42 ± 0.37 ^c^	14.72 ± 0.18 ^a^	25.15 ± 2.38 ^ab^
**OS7.5**	47.14 ± 2.17 ^a^	18.56 ± 0.28 ^d^	13.99 ± 0.81 ^a^	40.48 ± 5.20 ^c^

CO–control sample: pasta prepared with 100% of semolina flour, OS2.5, OS5 and OS7.5: pasta prepared with 2.5, 5, 7.5 g of onion skin powder/100 g of semolina flour.

Means with different letters in the same column are significantly different (P < 0.05), ± standard deviation for five replicate determinations.

The increase of redness parameter (a*) in raw and cook pasta enriched with OS showed significant increase (P < 0.05) compared to control samples (Table 4). The increased red intensity of samples was due to the color of the onion skin. It should be noted, that after cooking, color losses were detected because of the brighter and less red/brown colors of the enriched pastas. However, diffusion of the pigments into cooking water was slightly detected visually.

The yellow color of pasta is largely due to the presence of carotenoid pigments in semolina. Changes in yellow color intensity is described by the parameter b*. Similarly to the above, cooked pasta showed decreased b* value than control (37.72) to 13.99 (OS7.5), Table 4. Whilst there were no significant differences between yellowness (b*) of uncooked pasta samples, regardless of the OS concentration. Like with the reduction of a* (pasta less brown/red) after cooking, also value of parameter b* (pasta less yellow) can also be affected by partial removal of the colorant from pasta into water during cooking. In addition, pigment loss from semolina is resulted from degradation of some carotenoids during pasta processing through oxidation induced by lipoxygenase (LOX), resulting in pasta color loss [49].

The ΔE values was also determined to evaluate the color differences between the CO and the OS pastas. The ΔE values of pasta containing OS increased with increasing levels of OS in both cooked and uncooked forms (Table 4). Our results agree with those of Gallegos-Infante et al. [50] who found that an increase of Mexican common bean flour in pasta enhanced the color change. Values of ΔE higher than 44 were observed by Rajeswari et al. [4] in the study on quality characteristics of pasta enriched with onion powder and different hydrocolloids. Nowadays, the customers are accustomed to other than yellow pasta, they choose the ones with interesting colors to prepare more fancy dishes. In the case of products of the type like presented in this paper, dark coloring might be a positive feature as consumers identify it with high-fibre products, which is true in that case.

### Sensory evaluation

From the consumer’s point of view, the sensorial parameters of food products are critical points to ensure its acceptance. As previously mentioned, the quality of pasta, in particular: firmness, elasticity, cooking characteristics, are dependent upon the protein-starch network of the pasta product [40], also it is affected by all other ingredients incorporated into the pasta, such as OS (Table 1). Cooked samples fortified with different OS powder levels were evaluated for: appearance, color, taste, hardness, adhesiveness, springiness and overall acceptability (Table 5).

**Table 5:** Sensory characteristics of uncooked and cooked pasta fortified onion skin powder.

Pasta samples	Parameters						
	Color	Odour	Taste	Hardness	Adhesiveness	Springiness	Overall quality
**Uncooked pasta**							
**CO**	8.84 ± 0.37 ^d^	8.79 ± 0.57 ^c^	^-^	^-^	^-^	^-^	8.89 ± 0.32 ^d^
**OS2.5**	8.00 ± 0.87 ^cd^	8.47 ± 0.81 ^c^	^-^	^-^	^-^	^-^	8.79 ± 0.63 ^d^
**OS5**	7.58 ± 0.96 ^c^	8.47 ± 0.76 ^c^	^-^	^-^	^-^	^-^	7.32 ± 0.82 ^c^
**OS7.5**	6.63 ± 0.92 ^b^	8.31 ± 0.80 ^c^	^-^	^-^	^-^	^-^	6.96 ± 0.85 ^c^
**Cooked pasta**							
**CO**	8.47 ± 0.81 ^d^	8.37 ± 0.86 ^c^	8.68 ± 0.67 ^c^	8.79 ± 0.83 ^c^	8.26 ± 0.45 ^c^	8.79 ± 0.83 ^d^	8.47 ± 0.51 ^d^
**OS2.5**	7.42 ± 0.96 ^c^	8.21 ± 0.92 ^c^	8.42 ± 0.93 ^b^	7.53 ± 0.89 ^b^	6.63 ± 0.85 ^b^	7.63 ± 0.75 ^c^	7.26 ± 0.56 ^c^
**OS5**	4.73 ± 0.69 ^a^	6.95 ± 0.88 ^b^	5.42 ± 0.75 ^a^	5.53 ± 0.81 ^a^	5.42 ± 0.52 ^a^	5.63 ± 0.80 ^b^	6.05 ± 0.71 ^b^
**OS7.5**	4.89 ± 0.69 ^a^	4.58 ± 0.51 ^a^	4.63 ± 0.87 ^a^	5.00 ± 0.57 ^a^	5.00 ± 0.71 ^a^	4.47 ± 0.92 ^a^	4.95 ± 0.52 ^a^

CO–control sample: pasta prepared with 100% of semolina flour, OS2.5, OS5 and OS7.5: pasta prepared with 2.5, 5, 7.5 g of onion skin powder/100 g of semolina flour.

Means with different letters in the same column are significantly different (P < 0.05), ± standard deviation.

In general, all sensory characteristics were evaluated not lower than in the center point of the scale (5 = neither like nor dislike), indicating that pasta samples with OS were not disliked. All characteristics, except for OS2.5 appearance, were evaluated with scores lower than those of the pasta control. The taste and color of OS2.5 was comparable to CO, implying that such concentration of OS did not give any undesirable properties of pasta. The OS5 and OS7.5 samples were darker, and their color was less uniform. Application of higher content of the OS resulted in deterioration of taste. The specific aftertaste, described as “hay aroma”, gave lower scores of the taste in sensory analysis. An increase in content of the OS component (OS5, OS7.5) caused the pasta to have a softer structure. At the same time, its springiness and hardness decreased, resulting in a lowering of scores also for adhesiveness, which reached values below 6. The highest notes of overall quality was scored by OS2.5, similar to CO, which means that it could be the pasta that would also be accepted by a larger group of consumers. These results were different from those obtained by Rajeswari et al. [4] or Sayed et al. [41] who found that substitution of onion powder in 5 and 6%, had the best strand quality of pasta or noodles, respectively. These disagreements are probably due to differences in cultivars of onion and in particular, other ingredients used for the production of pasta and noodles.

## Conclusions

Results of this work represents a promising use of a by-product generated after onion processing, proposing onion skin as an ingredient in the manufacture of fortified wheat pasta. Our study lead to conclude that addition of onion skin powder to wheat pasta allows an evident improvement of several nutritional properties compared with non-supplemented pasta. We have demonstrated a noticeable increase in the total dietary fibre, total phenolic content, flavonoids and antioxidant capacity. From the technological point of view, the dry skin onion can be an additive that enriches the pasta. However, its quantity should be optimized every times depending on the quality of other materials used in the pasta production. This allows to obtain a product that is sensorial acceptable and at the same time has higher pro-health values.

The exploitation of by-products of fruit and vegetable processing as a source of functional compounds and their application in food is more often discussed in literature. Design of new food products should take account of complex matrix and their composition of bioactive principles. Also requires careful assessment of potential risks which might arise from isolated compounds recovered from by-products. Furthermore, investigations on stability and interactions of phytochemicals with other food ingredients during processing and storage need to be conducted. Consumer protection must have priority over economic interests, and health claims should be substantiated by scientifically sound and reliable studies.
